# Simulating the physiology of athletes during endurance sports events: modelling human energy conversion and metabolism

**DOI:** 10.1098/rsta.2011.0166

**Published:** 2011-11-13

**Authors:** Johannes H. G. M. van Beek, Farahaniza Supandi, Anand K. Gavai, Albert A. de Graaf, Thomas W. Binsl, Hannes Hettling

**Affiliations:** 1 Section Medical Genomics, Department of Clinical Genetics, VU University Medical Center, van der Boechorststraat 7, 1081 BT Amsterdam, The Netherlands; 2 Netherlands Consortium for Systems Biology, PO Box 94215, 1090 GE Amsterdam, The Netherlands; 3 Centre for Integrative Bioinformatics, VU University Amsterdam, De Boelelaan 1081A, 1081 HV Amsterdam, The Netherlands; 4 TNO Quality of Life, PO Box 360, 3700 AJ Zeist, The Netherlands; 5 Crosslinks BV, Willemskade 18c, 3016 DL Rotterdam, The Netherlands

**Keywords:** whole body modelling, metabolic pathways, heat transport, muscle power, skeletal muscle metabolism, mathematical model

## Abstract

The human physiological system is stressed to its limits during endurance sports competition events. We describe a whole body computational model for energy conversion during bicycle racing. About 23 per cent of the metabolic energy is used for muscle work, the rest is converted to heat. We calculated heat transfer by conduction and blood flow inside the body, and heat transfer from the skin by radiation, convection and sweat evaporation, resulting in temperature changes in 25 body compartments. We simulated a mountain time trial to Alpe d'Huez during the Tour de France. To approach the time realized by Lance Armstrong in 2004, very high oxygen uptake must be sustained by the simulated cyclist. Temperature was predicted to reach 39^°^C in the brain, and 39.7^°^C in leg muscle. In addition to the macroscopic simulation, we analysed the buffering of bursts of high adenosine triphosphate hydrolysis by creatine kinase during cyclical muscle activity at the biochemical pathway level. To investigate the low oxygen to carbohydrate ratio for the brain, which takes up lactate during exercise, we calculated the flux distribution in cerebral energy metabolism. Computational modelling of the human body, describing heat exchange and energy metabolism, makes simulation of endurance sports events feasible.

## 1. Introduction

The human physiological system is stressed to its limits during endurance sports competition events such as bicycle racing or marathon running. By computational modelling of the body during such challenging events, we can investigate adaptation under extreme stress, which is not only useful to understand the physiology of athletes but also of rescue workers, fire fighters and mountaineers, and others who must perform close to maximal capacity. Understanding the body's response under maximal stress may also help to understand disease processes. For instance, tissue hypoxia may be caused by great exertion rather than by ischaemia or tumour growth. Other examples are dehydration, high body temperatures caused by exertion rather than fever, and high blood lactate levels caused by physical exertion rather than hypoxia or shock [1].

Physical exercise affects human physiology at multiple scales. The physical work done by athletes is associated with force exertion, temperature changes in the whole body, sweat excretion and increased uptake of oxygen, water and food, all measurable at the whole body level. At the cellular scale, adenosine triphosphate (ATP) hydrolysis energizes the interaction of actin and myosin molecules in the sarcomeres of the muscle cells. The response of the body involves an extensive interplay between various organs. The heart, for instance, starts to pump more blood to transport oxygen and carbon substrates to the muscle and remove carbon dioxide, lactic acid and heat. The brain participates, among others, by regulating lung ventilation, but is challenged with increased lactic acid levels and higher blood temperature, potentially altering neuronal metabolism. The brain also controls muscle coordination and, importantly, determines the motivation of the athlete to perform maximally despite high brain temperatures, acidification, fatigue and sometimes even pain [2]. To prepare for the effort, the gut has to digest food to provide carbon substrates to the muscle cells via the blood stream. The whole body response is coordinated via hormones and the nervous system. In conclusion, modelling the human physiological system at macroscopic and microscopic scales during maximal athletic performance is an excellent test to demonstrate the possibilities of computational modelling for the physiome/virtual physiological human. The multi-scale aspects of modelling human physiology arise very naturally in this context.

Here, we will implement a whole body model for energy conversion and heat transport. The latter is very relevant because almost 80 per cent of the metabolic energy during exercise is converted to heat. We will couple this whole body model to equations for the motion and external power requirements during cycling. At the subcellular scale, we will look at models for intracellular events, which have a much higher biochemical and/or time resolution than the whole body model. We discuss the supply of metabolic fuel for exercise by nutrition. In addition, we introduce a model of metabolic flux distribution to predict how brain metabolism may deal with higher blood lactate levels. Metabolic events in the muscle cells are analysed at high time resolution with a model for the buffering of bursts of high ATP hydrolysis caused by the rhythmic muscle contractions during bicycle racing.

To demonstrate the applicability of whole body modelling to world class athletic performance, we simulate a mountain time trial in the Tour de France from Bourg d'Oisans to Alpe d'Huez. As the basis for our virtual cyclist, we use published physiological data [3] for Lance Armstrong, sevenfold Tour de France champion.

## 2. Implementing a whole body model of human energy conversion and heat transport

We will start with the description of a whole body computational model that describes the physiological system of an athlete performing external work at the macroscopic level. Although the model can be applied to various endurance sports, we will apply it to bicycle racing here.

The energy conversion relevant to propel the bicycle takes place in muscle. During cycling, less than a quarter of the energy obtained by burning nutrients with oxygen is converted to external work. The remainder of the energy is converted to heat, which is transported from the muscle by thermal conduction and circulatory convection. The blood also delivers oxygen and carbon substrates to the muscle and carries away the breakdown products, such as carbon dioxide and lactic acid, in addition to heat. The heat is distributed through the body by the blood and raises the temperature in all organs, including the brain and skin. The heat transported to the skin can be dissipated for a major part by evaporation of sweat. Which muscle groups are active depends on the type of exercise. Because of the specific localization of muscle activity and the extensive interplay between all organs, it is highly desirable that the whole body model contains multiple segments to represent distinct muscle groups and organs.

The whole body model can be viewed conveniently to consist of a controlled system, which contains metabolism and heat transport in blood and tissue, and a controlling system that regulates the response of the controlled system by neural and hormonal signals. Heart rate, cardiac output and lung ventilation increase under control of nerve signals from the brain. Sweat excretion and skin vasodilation are, for an important part, under central neural control. Therefore, the controller part of the model represents the action of the nervous and hormonal system on the heat transport in the controlled system, which consists of heat capacities, thermal conductance, tissue perfusion, etc.

A macroscopic model of human whole body physiology was described by Stolwijk & Hardy [4] and originally implemented on an analogue computer. It provides a set of equations that fulfil the requirements outlined above. The model for the analogue computer was modified and implemented on a digital computer by Stolwijk for the National Aeronautics and Space Administration [5]. The equations of the latter computational model were incorporated in the model presented here.

### (a) Representing compartments in the body: the controlled system

**Figure 1.**
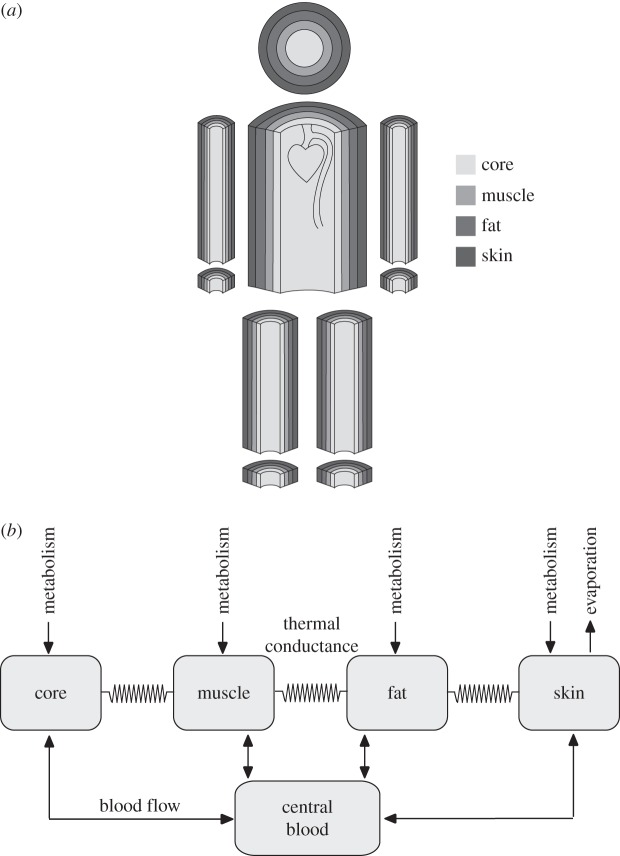
Scheme of the whole body model of heat transport. (*a*) The body is divided into six segments: head, trunk, arms, hands, legs and feet. Each segment consists of four layers approximated by concentric cylinders, except the head, which is a sphere. (*b*) Adapted from Stolwijk [5]. Heat is generated by metabolism, exchanged with the blood perfusing the layer and conducted to adjacent layers. Heat is dissipated from the skin by evaporation, but also by convection and radiation.

The controlled system of the body is divided into six segments in the model. The central segment of the body (the trunk) consists of the thorax and abdomen. Other segments represent the head, arms, hands, legs and feet (figure 1). Each of these segments is divided into four nodes: skin, (subcutaneous) fat, muscle and a core node. The central blood volume in the large blood vessels and cardiac cavities forms the 25*th* node. The cardiac output representing the total blood flow is partitioned to all the other 24 nodes and exchanges heat with the tissue. To each node, a certain mass, heat capacity and basal metabolic rate are assigned. For the head, for instance, the muscle mass is relatively small (about 0.4 kg), while in the legs, the muscle mass is large (about 10 kg). The core node of the head segment is relatively large (3 kg) because it represents the brain and the skull. The muscle nodes can contribute to external work by energy conversion from nutrients and oxygen. Below, we provide an outline of the model. Detailed equations for the model are given in the electronic supplementary material.

Metabolism leads to substantial heat generation in the muscle nodes, which is calculated from the total metabolic rate by subtracting the contribution made to external work. The generated heat divided by the heat capacity of each node gives the potential rate of increase of the local temperature. Each node receives a fraction of the total cardiac output, and heat generated in the tissue is exchanged with this blood flow. Heat is also transported between the adjacent nodes by heat conduction through tissue. From the surface of the body inward, layers of skin, fat and muscle are encountered until the core of the segment is reached. This is represented by the four nodes per segment. The simple equation for conductive heat transfer TD from node *N* to node *N*+1 is
2.1
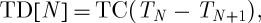
where TC is the thermal conductance, which is multiplied by the temperature difference between the nodes. For the skin nodes, this equation is replaced by an equation that takes radiative, convective and evaporative heat loss to the environment, including the effects of humidity and ambient temperature, into account (see the electronic supplementary material). The convective heat transport BC[*N*] from a node by blood flow BF is given by
2.2
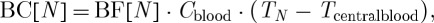
with *C*_blood_ the heat capacity of blood. This equation is simple because the temperature in blood flowing through small arteries and arterioles becomes almost equilibrated with tissue, even before it reaches the capillaries [6].

The rate of change, d*T*/d*t* in temperature of node *N* is given by
2.3
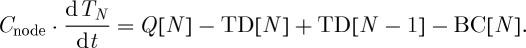
The metabolic heat generation *Q*[*N*] consists of a basal heat production rate QB[*N*] plus a potentially large additional heat production in muscle during exercise. *C*_node_ is the heat capacity of the node. Even if a node has low *Q*[*N*], its temperature may rise considerably because of heat conduction from adjacent nodes and heat transfer from the blood. Heat may, for instance, be transferred from leg muscles to the brain and skin via the blood.

A large quantity of heat is transported to the environment via the skin by radiation, convection and evaporation of water. Water is excreted from the sweat glands under central and local control. The skin also loses heat by convection, which is very much increased by high air velocity. This velocity is not only caused by the wind, but also by the motion of the cyclist. A small fraction of heat is given off to air in the airways and lungs by conduction and evaporation. Detailed equations are given in the electronic supplementary material.

### (b) Sweating, shivering and increasing cardiac output: the controlling system

The body senses the local temperature in various locations, among others in the brain and in the skin. Signals from the sensors are sent to the brain. If temperature is increased, the central controller sends signals to cause skin vasodilation and sweat production. If body temperature falls, signals are generated to produce heat by shivering and to constrict the blood vessels that control skin blood flow. Relatively little is known about the nervous mechanisms that sense tissue temperature and which form the control system in the brain, generating efferent signals to effector mechanisms that control heat generation and transport in the body [7]. Peripheral nerve signals have been measured that are responsive to temperature, and the anterior hypothalamus region of the brain is sensitive to temperature changes. However, there is insufficient information about the nervous regulation of temperature to model the controller in mechanistic detail.

Therefore, in the computational model, a simple black box approach was chosen: the deviation of node temperatures from a reference temperature constitutes the input to the central controlling mechanism [5]. In particular, the central controller uses the temperature of the brain and of the skin nodes as input. Under thermoneutral conditions, the error signal is assumed to be zero, and no efferent signals are generated to stimulate sweating, skin vasoconstriction, vasodilation or shivering. It is unlikely that the temperature is explicitly compared with a set point in the real body [7], but the simple black box formulation adequately describes thermoregulatory responses.

The equations describing the controller were parametrized based on extensive measurements of the relation between input signals (brain and skin temperatures) and output signals (rate of sweat evaporation, blood flow to the skin) [4,5]. Therefore, the simple equations describe the reaction of the human body to temperature change adequately, based on a solid experimental basis. The equations and parameter values for the control of vasodilation, vasoconstriction in the skin, sweating and shivering are given in the electronic supplementary material.

### (c) Representing voluntary control of muscle activation

For an athlete who tries to win a race, not only the physical work capacity counts, but also the motivation to go to the limits of performance despite exhaustion and perhaps even pain. The key parameter for the physical capacity is the maximal oxygen uptake of the athlete that can be measured in the laboratory. However, during a long effort, oxygen uptake cannot be sustained at the same level as during a relatively short test in the laboratory. The available mechanical power tends to show a downward trend during a long race [2]. Because the complex neural processes that determine the sensation of fatigue and exhaustion and the voluntary control of muscle contraction during exertion are insufficiently known, in the present model, these mental processes are enormously simplified by a ‘brain push’ factor, which gives the fraction of maximal oxygen uptake that will be used by the athlete.

### (d) Integrating the model

The ordinary differential equations were implemented and integrated in R computing language using the lsoda routine, which is based on algorithms by Hindmarsh [8] and Petzold [9]. R is a high-level computer language and computational environment, which is open source and provides a large collection of open-source analysis tools [10].

## 3. The power required for bicycle racing

The external mechanical power available to increase and maintain the speed of the cyclist–bicycle system is delivered by the muscle nodes in the computational model of the cyclist. Physical power equals the force propelling the bicycle times the velocity. The opposing forces that must be overcome to propel the bicycle are (i) the frictional force of the air (wind drag), (ii) the frictional force of the tyres on the road (rolling resistance), (iii) the component of the force of gravity, which is parallel to the road surface when climbing, and (iv) the force used to accelerate or decelerate. The external power delivered by the cyclist's muscles is balanced by the power of the opposing forces. A voluntary decision to exert more force is associated with more external power and may lead to acceleration.

Equations describing the components of the resistance to motion have been derived and their coefficients measured. The rolling resistance (*R*_r_) is a force (in N) given by
3.1
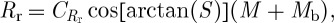
where *S* is the gradient of the road (i.e. vertical distance/horizontal distance), *M* is the mass of the rider and *M*_b_ is the mass of the bicycle [11]. This expression gives the normal force exerted by the combined mass multiplied by a constant *C*_*R*r_ that depends on wheel radius and road surface and is approximately inversely dependent on tyre pressure. The friction of the chain and other moving parts of the bicycle are usually negligible. The measured values of the parameters in the power equations and further equations are given in the electronic supplementary material.

The air resistance given as a force in N is
3.2
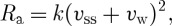
where *v*_ss_ is the speed of the bike relative to the road and *v*_w_ is the speed of the head wind relative to the road (negative for a tail wind) [11]. The coefficient *k* is proportional to the frontal projected area and air density. This dependency is given quantitatively in the electronic supplementary material.

If the road is sloping, the component of the weight of rider plus bicycle parallel to the road, *F*_g_, is needed to account for gravity. An additional force *F*_acc_ can be used to accelerate. The instantaneous power needed to propel the cyclist at a certain moment in the race equals the instantaneous velocity multiplied by (*R*_r_+*R*_a_+*F*_g_+*F*_acc_). The power to propel the bicycle is balanced in the model by the power of muscle work made available from metabolic processes. This power is determined not only by the cyclist's physical capacity, but also by the motivation to win and the pacing strategy that is consciously followed (e.g. starting as fast as possible versus somewhat slower to save energy for later). The motivational factors are represented by the brain push factor mentioned above. The balance of the power required and delivered by the cyclist determines whether he accelerates, decelerates or maintains a steady speed. The acceleration is integrated to obtain the speed of the cyclist, which determines the road distance covered. The equations in the computational model are given in the electronic supplementary material.

## 4. Simulating a time trial to Alpe d'Huez

Here, we apply the model to simulate a mountain time trial in the Tour de France. The time trial starts in the town of Bourg d'Oisans at an altitude of about 720 m above sea level, and ends after 15.5 km in the town of Alpe d'Huez at an altitude of 1850 m. The conditions are similar to the mountain time trial in the Tour de France on 21 July 2004 [12,13], although we emphasize that exact correspondence to the parameters of the time trial and the cyclist could not be established. The altitude of the road at 1 km intervals was obtained from published profiles [14]. From these altitudes, we calculated the slope of the road with a 1 km resolution. The climb starts 1.5 km from the start and the slopes vary between 8 and 11 per cent for three quarters of the distance covered. For the anthropometric and physiological characteristics of the rider in the model, we use published data for Lance Armstrong [3], who won the stage with a time of about 39 min 41 s, more than 1 min faster than Jan Ullrich, who finished second.

In the simulation, the body mass of the rider was estimated to be 72 kg during the race, and his maximal oxygen uptake determined in the laboratory is 6.1 l O_2_ per minute, which is the highest value recorded during repeated laboratory tests for this individual [3]. An important parameter determined in the laboratory is the Δefficiency, which is defined as the increase in work accomplished on the bicycle ergometer per unit time divided by the energy expended during the same time. The energy expended was calculated from the measured oxygen uptake and reflects the energy available from the oxidation of metabolic fuel, mainly carbohydrate and fat, with oxygen. The work accomplished per unit time is measured by determining the mechanical power delivered by the cyclist to the bicycle ergometer. The increase in oxygen consumption from the resting level the cyclist manages to accomplish is multiplied by the Δefficiency, 0.231 in this case, and by the total energy expended per amount of oxygen to calculate the energy available for propelling the cycle–cyclist system.

For the hypothetical case that the cyclist would be able to use 100 per cent of his maximal oxygen uptake, as measured during a short bout of intense exercise in the laboratory at lower altitude, the simulation predicts that the time to complete the 15.5 km up to Alpe d'Huez is 38 min 31 s. Here, it was assumed that the decrease in barometric pressure during the climb to 1850 m leads to reduced air resistance because of lower air density. For two important reasons, it is not realistic to assume that 100 per cent of maximal oxygen uptake can be sustained. Firstly, it is generally found that cyclists can sustain less than 100 per cent oxygen uptake if the effort lasts longer than 10 min [11]. The second reason is that the progressively lower oxygen partial pressure in the air at higher altitude will lead to lower maximal oxygen uptake. The relative reduction of maximal oxygen uptake with a lower concentration of oxygen in the air exists in untrained subjects, but is even stronger in trained athletes [15], where an average decrease of about 13 per cent in oxygen uptake is found if oxygen partial pressure in the air falls by 24 per cent. At an altitude of 1850 m, oxygen partial pressure is expected to be decreased by about 20 per cent relative to sea level. If the simulation includes this effect of hypoxia on oxygen uptake, the predicted time becomes 41 min 22 s.

The actual time of Lance Armstrong in 2004 was about 39 min 41 s. This time is found in the simulation of the virtual cyclist if 96.5 per cent of the maximal uptake of 6.1 l O_2_ per minute can be sustained during the climb to Alpe d'Huez, assuming no further reduction owing to altitude hypoxia. A sustained fraction of 0.965 during about 40 min is very high relative to trained (but non-professional) cyclists [11]. To obtain the time realized by Lance Armstrong during the mountain time trial, it is necessary to assume an oxygen uptake of about 5.9 l min^−1^ for the virtual cyclist, despite the reduced oxygen partial pressure encountered when climbing from 720 to 1850 m. Given that professional cyclists often aim for peak performance at the time of decisive races, it is possible that a higher value of maximal oxygen uptake (VO_2_max) was valid during the Tour de France in comparison with the value of 6.1 l O_2_ per minute reported for the laboratory tests, which were carried out in earlier years.

Assuming a very high will power of the simulated cyclist such that he is able to maintain 95 per cent of the maximal O_2_ uptake and assuming that the reduction of oxygen uptake by altitude hypoxia is as measured by Ferretti *et al.* [15] for trained endurance athletes, we estimate that the maximal oxygen uptake measurable at sea level should have been 6.74 l O_2_ per minute to complete the time trial in 39 min 41 s. We emphasize that it was not possible to obtain all parameters for the cyclist and the time trial with great accuracy, implying that we most probably obtained incomplete correspondence between the virtual cyclist in the model and Lance Armstrong during the 2004 time trial. Our estimates of oxygen uptake must, therefore, be treated with great caution. For instance, the sensitivity of real professional cyclists to altitude hypoxia may differ from that assumed in the model. We assumed that the simulated cyclist would be able to maintain 95 per cent of maximal O_2_ uptake, i.e. the brain push factor mentioned above. If this value is actually higher or lower, the estimate of the maximal O_2_ uptake corresponding to the performance during the time trial will have to be adapted.

**Figure 2.**
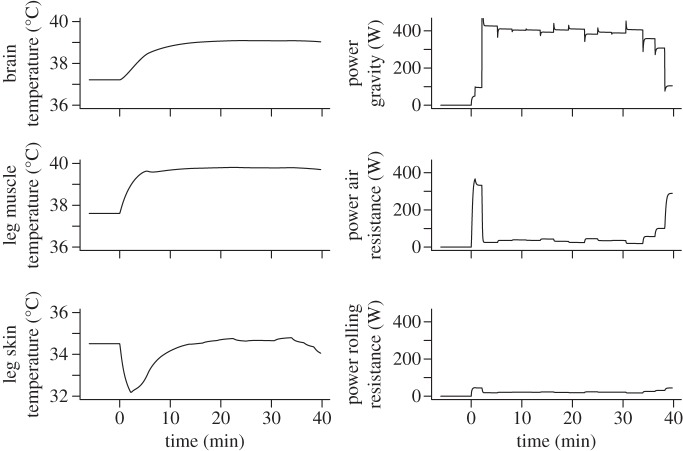
Predictions of the main fates of metabolic energy converted in the muscle during the mountain time trial. (*a*) The increasing temperatures of leg muscles and brain reflect heat generation. The increasing air velocity across the skin during the time trial leads to significant cooling, but this effect becomes less at steeper slopes, where speed is diminished. (*b*) Power (in Watts) expended against gravity, air resistance and rolling resistance. The sharp spikes in the power against gravity are caused by the sharp transitions to different grades caused by the low resolution (1 km) by which the slope of the road to Alpe d'Huez was known to us: the cyclist enters a section with a steeper grade with high velocity and is subsequently slowed down by the increased opposing gravitational force. The cyclist finishes after 15.5 km in 39 min 41 s.

Simulation results for tissue temperatures and power dissipated by air resistance, rolling resistance and gravity for the cyclist with 6.74 l min^−1^ maximal O_2_ uptake are given in figure 2. About 77 per cent of metabolic energy was measured to be converted to heat in this Tour de France champion [3], which leads to significant warming of leg muscles and brain to over 39^°^C.

The cyclist who finished second in 2004 was reported to be 5 cm taller than Lance Armstrong. If the body height of the virtual cyclist is increased from 179 cm to 184 cm, the model simulation predicts that the time needed for the time trial becomes about 3 s longer. If the total weight of the rider and bicycle is 2.4 kg larger, with muscle mass and oxygen uptake constant, the predicted time becomes 40 min 42 s, which is about the time of Jan Ullrich, who finished second in 2004. Larger body weight may, among others, be caused by greater fat or skeletal mass. The slowing effect of the added weight is for the largest part caused by the increase in the component of the gravitational force parallel to the road surface. However, smaller effects are due to the component of the force normal to the road surface increasing the rolling resistance, and to increased air resistance owing to increased size of the body. This illustrates that small differences in body size can have significant impact on athletic performance.

## 5. Validating the whole body model

The parameters of the controlled (e.g. metabolism and heat transport) and controlling systems (neural and hormonal regulation, see detailed explanation above) were measured on human subjects [4,5]. For new subjects with other characteristics than the original study group, the model may reproduce steady-state thermoregulatory responses less accurately. The transient response to sudden changes in thermal stress forms a more stringent test than steady-state responses. To this end, an increase in air temperature from 30^°^C to 48^°^C for 2 h was simulated. The temperature of the walls was equilibrated with the air temperature during the experiment. The response of average skin temperature was measured to increase from 34^°^C to 36.5^°^C [16,17], while the model predicted an increase from 33.4^°^C to 36.3^°^C. Both the present model implementation and the original model of Stolwijk [5] predict a temperature increase in the core of the trunk, which was significantly smaller than the increase in rectal temperature measured in a group of young subjects [16].

We further simulated experiments by Nybo *et al*. [18], where subjects started to exercise at 174 W on a bicycle ergometer at 20^°^C ambient temperature. In the simulation, the brain temperature increased from 37.28^°^C to 37.96^°^C after 45 min. In the experiments, these numbers were 37.2^°^C and 38.0^°^C for the jugular venous temperature at the base of the cranium, which is close to the brain temperature. The time course of brain temperature was quite similar in the simulation and Nybo's experimental study.

The model equations of Stolwijk & Hardy [4] are quite extensive. Many measured trends and variables are predicted remarkably well, although significant deviations from the measurements are also found [5,16]. Indeed, Stolwijk discussed a number of shortcomings of the model [5]. Although the model in most cases predicts the right order of the response, it is desirable to further validate and improve this extensive model. Parameters may be fine tuned to fit special groups of subjects, for instance, trained professional athletes. The model parameters are representative of a group of human subjects studied in the laboratory, but were not yet adjusted for any individual, in this case, a world class cyclist. The parameters can, for instance, be further fine tuned by imaging the body of the modelled individual using magnetic resonance imaging and obtaining the VO_2_max closer in time to the real time trial. The frictional resistance of the bicycle and air resistance in a wind tunnel could also be measured. It would then be possible to test the model by measuring the speed and delivered power of the cyclist and the slope of the road at greater resolution than has been possible here. Heart rate, skin and rectal temperatures could be measured non-invasively during a time trial. By weighing the body as well as fluid and food intake, the net evaporation during the time trial can be assessed.

What is still hard to quantify is the athlete's determination to voluntarily go as fast as possible. The effort made by an athlete will vary during a race: often, it is very high at the start of a time trial, falling quickly and sometimes showing periodic behaviour [2,19]. In short, although the model shows responses that are in line with existing experimental measurements for groups of subjects, the model parameters may be adjusted to better approach individual characteristics and may be tested in the field on individuals during a time trial out of competition.

## 6. Time-resolved model of events at the muscle cell level

In the whole body model discussed so far, metabolic processes inside muscle cells were not modelled in detail. In this section, we focus on how energy metabolism at the cellular level can be described and predicted by means of mechanistic modelling with much higher time resolution than feasible for the whole body model. Endurance exercise requires permanent energy supply to enable contractile work in skeletal muscle. However, the amount of ATP in muscle cells is merely sufficient to energize two or three maximal contractions in skeletal muscle under normal conditions [20]. Therefore, aerobic and anaerobic ATP production must be tightly coupled with the energetic demands of the muscle cell. Creatine kinase (CK) enzymes provide a buffer between energy producing and consuming pathways. CK facilitates additional energy storage in the form of phosphocreatine (PCr) by catalysing the reversible transfer of a phosphate group to creatine (Cr),

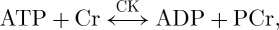
where ADP stands for adenosine diphosphate. Previously, long ago, PCr was suggested to maintain intracellular ATP homeostasis during muscle contraction [21]. The discovery of two distinct isoforms of CK, Mi-CK in mitochondria and MM-CK in myofibrils, led to the hypothesis of a role of CK as an energy shuttle from ATP producing to ATP consuming sites across the mitochondrial outer membrane (MOM) [22]. In previous work, we investigated the functions of CK using a mathematical model of energy metabolism in a cardiac myocyte integrated with data from various scales [23–25]. Our model predictions emphasized the role of CK in damping the rate of oxidative phosphorylation and ATP and ADP levels during high-amplitude bursts of ATP consumption during the cardiac cycle. In this section, we show that our model can describe experimental data from human skeletal muscle collected during cycling. As a result, our model predicts buffering of dynamic ATP synthesis and metabolite levels.

**Figure 3.**
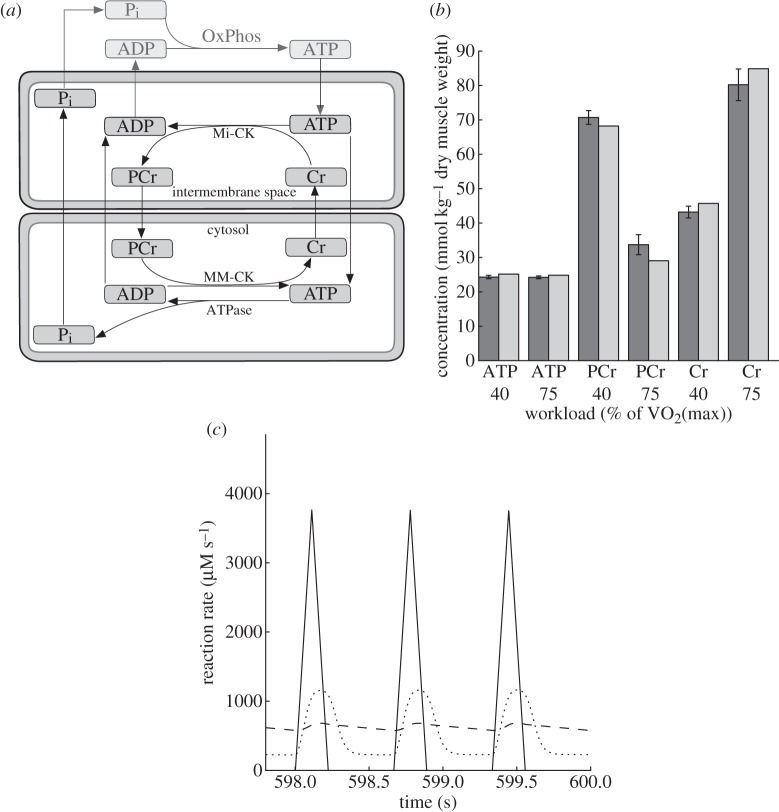
(*a*) Scheme of the model of the creatine kinase (CK) system. Cell and mitochondrial membranes are indicated. Cr, creatine; PCr, phosphocreatine; Pi, inorganic phosphate; OxPhos, oxidative phosphorylation. (*b*) Steady-state metabolite concentrations at the end of a 600 s simulation compared with data from cycling exercise experiments at different submaximal workloads [26]. For conversion of metabolite concentrations between model (μmol l^−1^ cell water) and data (mmol kg^−1^ dry weight), we assumed an intracellular water content of 3 l kg^−1^ dry muscle weight [27]. (*c*) Model prediction of the ATP synthesis time course at 100% (dashed line) and 2% (dotted line) of normal CK activity at a workload of 40% of VO_2_max. The forcing function of pulsatile ATP hydrolysis is plotted as a solid line. Note that the last 2 s of a simulation over 600 s are shown ensuring a steady state. (*b*) Dark grey, data; light grey, model prediction. (*c*) Dashed line, ATP synthesis, 100% CK; dotted line, ATP synthesis, 2% CK; solid line, ATP hydrolysis.

The model consists of four major components in two compartments that capture the key elements of the CK system (figure 3*a*): (i) mitochondrial ATP production by oxidative phosphorylation (OxPhos), (ii) high-energy phosphate transfer by Mi-CK in the mitochondrial intermembrane space, (iii) the MM-CK reaction in close vicinity to myofibrils, and (iv) ATPase activity, represented by a forcing function of ATP hydrolysis simulating pulsatile energy consumption of the contracting muscle.

Parameter values for Mi-CK reaction, kinetics of ATP production and MOM permeability to metabolites were collected from van Beek [23]. Kinetic constants for skeletal muscle MM-CK were reported in Vicini & Kushmerick [28] based on earlier measurements [29]. To fine tune all 22 kinetic model parameters, we use data from an experiment in which 10 well-trained subjects performed an incremental challenge on a bicycle ergometer at submaximal workloads of 40 and 75 per cent of their maximal aerobic capacity (VO_2_max). After each workload challenge, a muscle biopsy from the quadriceps femoris was taken and analysed for intracellular concentrations of ATP, PCr and Cr (figure 3*b*) among others [26]. The [H^+^] concentration reported for this study ranged from 0.89 10^−7^ M at rest to 1.20 10^−7^ M at 75 per cent of VO_2_(max) [26], and was taken into account in the calculation of the CK reaction [30]. Maximal aerobic work rates were excluded from this analysis because anaerobic metabolism starts to play a major role and is not yet included in the model.

To estimate the rate of ATP hydrolysis powering contractile work in leg muscle, we use the average VO_2_max of 4.1 l min^−1^ of the subjects in the bicycling exercise [26], measurements of aerobic capacity in skeletal muscle [31], and assume 10 kg active leg muscle mass for well-trained athletes [32]. Further, we assume a P/O ratio of 6, meaning that six molecules of ATP are produced per molecule of oxygen consumed [32]. From this, we estimate an average ATP hydrolysis flux, *J*_ATP,hyd_, of 628 and 1175 μM s^−1^ for 40 and 75 per cent of VO_2_max, respectively. Parameter estimation was done with the downhill simplex algorithm implemented in the SloppyCell modelling environment [33]. Because the kinetic parameters were taken from solid experimental data [29], we constrained these parameters based on their reported measurement error using Bayesian prior terms added to a least-squares cost function. Parameters for which no measurement error was reported in the literature were constrained by a default prior term as in Hettling & van Beek [25]. Taking this *a priori* information into account yields more robust parameter values [34]. Results of the parameter fit to the metabolite concentrations for two experimental conditions are shown in figure 3*b*. The model predicts a decline of PCr, the high-energy phosphate store, with increasing workload, while ATP levels stay relatively constant. The model predictions for PCr, Cr and ATP are relatively close to the measured values, despite the fact that the kinetic parameters in the model were kept close to their reported literature values by constraining them with their measurement error using Bayesian priors.

A limitation of the present model analysis is the absence of glycolytic ATP formation. Tissue lactate levels were reported to be substantially elevated going from rest to 75 per cent of VO_2_(max) [26] at the time point of the phosphate metabolite measurements (figure 3*b*). Because lactate production may change at later stages of exercise, the incorporation of dynamic changes in lactate production and intracellular pH in future modelling work is desirable.

For many professional cyclists, mechanical efficiency is maximized at pedal cadences around 90 r.p.m. [35]. In order to simulate alternating energy usage of the leg muscle at this frequency, we used a pulsatile forcing function of ATP hydrolysis. The shape of the ATP hydrolysis pulse is triangular (figure 3*c*), as used previously for a beating heart [23]. The leg muscle is assumed active during one-third of the pedal stroke cycle. This time course and the fractional value of one-third is close to predictions based on biomechanical calculations and to electromyographic measurements of the activity pattern in the quadriceps muscle during one pedal stroke [36]. Given this triangular activation curve during one-third of the total cycle, the peak value of the ATP hydrolysis rate is sixfold the average ATP rate, which in turn is estimated from oxygen consumption data.

To investigate the effectiveness of CK in buffering these pulses in ATP demand, we simulated conditions of normal and impaired enzyme activity (figure 3*c*). With normally active CK, the amplitude of ATP synthesis is almost fully buffered. Blocking CK by 98 per cent results in a ninefold increase of the amplitude of the ATP synthesis oscillations from 106 to 938 μM s^−1^. These results demonstrate the importance of the CK system in maintaining cellular ATP homeostasis and buffering of high amplitudes in mitochondrial ATP production in skeletal muscle during cycling.

## 7. Detailed models of carbon metabolism in the cell

Energy for muscle contraction is derived from the oxidation of carbohydrates and fatty acids derived from the food. The energy is used to synthesize ATP. The pathways for the metabolism of these carbon substrates may be analysed with computational models, which represent the biochemical reaction networks consisting of enzymes and of metabolites. A certain metabolite can be produced by one particular type of enzyme and be used by the next enzyme in the metabolic network. The enzyme content determines the maximal rate at which a reaction can take place. The precise distribution of the flow of carbon in the intracellular metabolic pathways can be predicted by models of interconnected metabolic reactions. The calculation of the flux distribution in the network resembles calculations based on Kirchhoff's law in electrical circuits: the amount of material flowing into a node in the network must equal the amount of material flowing out. For instance, if reaction 1 produces a certain metabolite X at flux *V*_1_, while reactions 2 and 3 consume one and two molecules, respectively, of X at fluxes *V*_2_ and *V*_3_, the balance equation for metabolite X is
7.1
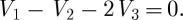
Additional constraints on the flux distribution can be derived from biochemical knowledge. The enzyme cytochrome oxidase that transfers electrons to oxygen operates, for instance, in an irreversible way and will never produce oxygen. The first enzymatic reaction in the glycolytic pathway after the uptake of glucose in the cell is an irreversible reaction catalysed by the enzyme hexokinase,

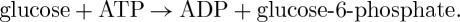
Some, but not all, reactions in the biochemical networks have such an irreversibility constraint. Measurements on input and output fluxes from cells or measured capacities of enzymes may form additional constraints. In this way, flux balance analysis (FBA) [37] derives the possible distribution of metabolic fluxes in the network. If some of the most relevant central pathways of metabolism are included (e.g. the glycolytic pathway, the pentose phosphate pathway, the tricarboxylic acid cycle and transamination of carbon skeletons to amino acids), a model of flux distribution often contains more than 50 enzymatic reactions.

### (a) A computer package to calculate flux distributions

Most of the relevant reactions in the cell can be found in databases of metabolic pathways. In order to facilitate the building of metabolic pathway models, we developed the computer package BiGGR, which, among others, downloads the reactions from the Biochemical, Genetic and Genomic (BiGG) knowledgebase of metabolic reconstructions, which contains several thousand manually curated enzymatic reactions for the human system [37]. The name BiGGR is derived from the BiGG database [37,38], while R stands for the R programming language. The R and Bioconductor framework are widely used among the genomics community [39]. In BiGGR, the user can choose the reactions for the model analysis. The computer program then builds the stoichiometric matrices from the chosen set of reactions describing all balance equations such as equation (7.1). The first matrix contains the set of linear equations, where metabolites are represented by the rows and reactions are represented by the columns. Linear inequalities that represent the constraints are represented by a second matrix. The systems of equations for metabolic systems are usually underdetermined [37]. Despite this situation, it is often possible to derive the range of values that are feasible for the metabolic reactions. Sampling the flux space, for instance, by simple uniform sampling, makes it possible to explore which combinations of fluxes are compatible with the constraints. Alternatively, one can sample a probability distribution in flux space by a Markov chain Monte Carlo procedure: the probability that a point in flux space is included in the sample is proportional to the probability that this point represents measured data. If one can design a plausible linear combination of fluxes, which forms a cost function to be minimized (e.g. the total production of metabolic waste products) or forms a gain function to be maximized (e.g. the production of ATP), it is often possible to determine a unique flux distribution although the system of linear equations is underdetermined.

BiGGR facilitates FBA, building on a range of linear optimization routines, which were already available for R [40]. In addition, BiGGR also attempts to automatically visualize the metabolic pathways using a ‘hypergraph’ format (P. Murrell 2009, personal communication), plotting the numerical results of a flux analysis on the pathway graph.

### (b) Simulating brain metabolism during exercise

Glucose is the main substrate for fuelling cerebral metabolism under normal physiological conditions when blood lactate concentration remains low. The ratio of O_2_ uptake/glucose uptake is close to 6 [41]. During maximal exercise, glucose uptake increases and the brain may also use arterial lactate released by the muscles [42]. However, oxygen uptake does not increase proportional to carbohydrate uptake, indicated by a cerebral metabolic ratio (O_2_ uptake/(glucose+1/2 lactate) uptake), which falls below 3. The fate of carbohydrate in the brain during exercise remains unknown [43]. With nuclear magnetic resonance spectroscopy, no glucose or lactate accumulation is found and the capacity to synthesize glycogen may be too low to account for non-oxidized glucose and lactate [42,44]. Immediately after maximal exercise, a small increase of free fatty acid release is detected, while no change was measured for other substrates including glutamine, glutamate and alanine [45].

To investigate whether excess carbohydrate uptake can be explained by fatty acid synthesis, we developed a computational model that represents pathways of central metabolism in the brain and simulated the fluxes of glucose and lactate metabolism during rest and maximal exercise. Given the high lipid content of the brain, this is a plausible hypothesis. The reactions in our model comprise central energy metabolism and fatty acid synthesis in their appropriate compartments (cytosol and mitochondria), and metabolite transport across the mitochondrial membranes. The inclusion of the malate-aspartate and glycerol-3-phosphate shuttles allows for transport of reducing equivalents between the cytosol and mitochondria. Amino acid synthesis of alanine and glutamate/gamma-amino butyric acid (GABA) cycling were included. Reactions are taken from the BiGG database, and measurements of substrate uptake in the brain during rest and exercise were taken from Quistorff *et al*. [44]. We use the linear inverse modelling routines [40] linked to the package BiGGR for the calculations. The system is assumed to be in a steady state, with a cost function to maximize ATP production. A list of full reactions and constraints used in this model, input and output fluxes assumed for the cell, as well as the flux distribution resulting from the analysis are given in the electronic supplementary material.

**Figure 4.**
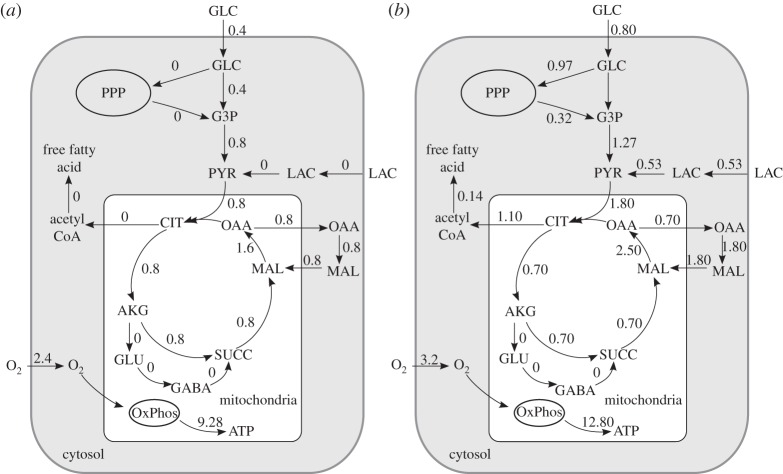
Models of carbon metabolism in brain. (*a*) Simulation of cerebral energy metabolism at rest and (*b*) during 15 min maximal exercise. Substrate uptake is from Quistorff *et al*. [44]. Flux values are in mmol min^−1^ for the whole brain. PPP, pentose phosphate pathway; OxPhos, oxidative phosphorylation; GLC, glucose; PYR, pyruvate; LAC, lactate; GLU, glutamate; GABA, gamma-amino butyric acid; MAL, malate; OAA, oxaloacetate; SUCC, succinate; AKG, alpha-ketoglutarate; CIT, citrate; CoA, coenzyme A; G3P, glyceraldehyde-3-phosphate. Please note that for clarity not all reactions are shown and the metabolites are sometimes balanced by reactions that are not plotted. There is, for instance, a small backflux towards glucose in the upper part of the glycolytic chain in (*b*).

At rest with no lactate present, metabolism of 0.4 mmol min^−1^ glucose in the whole human brain is in agreement with the O_2_/glucose ratio and yields a maximal mitochondrial ATP synthesis of 9.28 mmol min^−1^. No flux through the pentose phosphate pathway is predicted (figure 4*a*). During 15 min of exercise, glucose uptake doubles, and, in addition, 0.53 mmol min^−1^ lactate and 3.2 mmol min^−1^ oxygen are taken up. We hypothesize that given the low ratio of oxygen to glucose and lactate uptake, the carbon in these carbohydrates may be used to synthesize and store fatty acids. We calculate that 0.14 mmol min^−1^ of fatty acid (equivalent to palmitate with 16 carbon atoms) must be synthesized in the brain to balance glucose and lactate uptake with the limited availability of oxygen to oxidize carbon (figure 4*b*). The pentose phosphate pathway is now activated to produce nicotinamide adenine dinucleotide phosphate, which is required for the synthesis of free fatty acid. The result above suggests that fatty acid synthesis, perhaps followed by incorporation into lipids that are abundantly present in the brain, is a possible pathway for the excess carbon. Future experiments to measure storage of cerebral fatty acid, possibly using stable isotope labelling of precursors [24,46], allow this hypothesis to be tested.

## 8. Future directions

The model approaches discussed address various spatial, temporal and biochemical resolutions and are conceptually complementary. However, they were not yet linked in an integrated computational framework and executed simultaneously. For the buffering of the ATP hydrolysis peaks in the CK model, we calculated the amplitude of ATP hydrolysis offline. Yet, it seems feasible to couple the CK model to the whole body model. In that way, the energy turnover in the leg muscles would be coupled directly to the amplitude of ATP hydrolysis during cyclical work on the bicycle. The levels of inorganic phosphate and the maximal ADP concentration during the cycle could be used to indicate the fatigue status of the muscle and might be used to determine the obtainable level of exertion. The minimal concentration of ADP may additionally indicate the risk of oxygen radical formation because of low stimulation of the mitochondria.

To develop computational models of endurance sports performance further, it might be very efficient if a framework existed where the modelling approaches at various scales could be seamlessly integrated. In addition, if one builds and links models of metabolic pathways, it is very efficient if one can integrate biochemical reactions available from databases. A prerequisite for such an exchange of reactions is a uniform standard nomenclature and symbols for metabolites, enzymes and other entities in physiological models. For the present work, we used the nomenclature of the BiGG database [38], but it is clear that an ontology which is generally accepted by the biomedical research community or enforced by leading journals is needed.

Given the extensive research literature on sports physiology and the vast knowledge on basic biology, it is timely to start to develop quantitative models of human physiology. Quantitative integration of physiological and biochemical knowledge in computational models may facilitate the understanding of human performance, and may increasingly become a central research tool for integrative physiology. Because professional world class athletes push their performance to the very limits, model analysis of their performance during sports events may provide a unique window on human physiology. By demonstrating the application of virtual physiological modelling to sports or space travel, we may arouse the interest of the general public and young scientists. The benefits for medical applications and enhanced safety surveillance of athletes will no doubt follow.

Our model mainly predicts the maximal VO_2_ value achieved during the race. The result suggests that VO_2_max explaining the performance during the race is higher than the maximum found in laboratory tests earlier in time. Athletes use all sorts of methods to prepare optimally for the decisive race. Many athletes expose themselves to low oxygen concentrations, for instance, by sojourn at high altitude or by breathing gas mixtures with decreased oxygen content during sleep, to increase natural erythropoietin production. The winner of the 2004 time trial to Alpe d'Huez may have used hypoxic exposure as reported in Coyle [3]. In addition, athletes use training schemes aimed at obtaining peak performance during the decisive stages of the event.

Our model does not yet contain oxygen transport to make it possible to analyse oxygen delivery and uptake. It is, therefore, not yet possible to analyse the effects of illegal (recombinant erythropoietin and autologous blood transfusion) or legal methods (hypoxic exposure) to support increased oxygen uptake with our model, but this may be an interesting future expansion of the model. An important aspect of preparation for a long endurance sports event is optimal nutrition to supply sufficient carbon fuel for long, intense exercise. The effect of nutrition on glycogen and fat stores in the body and the interaction of carbohydrates and fat during exercise are aspects that require extensive model analysis in the future. The glycaemic index or the glycaemic load, which describe the effect of various carbohydrate formulations can be incorporated in the models [47]. The interaction of protein given in combination with carbohydrates may also be incorporated in the model description of carbon substrate handling before, during and after exercise [48,49]. An overview of nutritional support for athletic performance is given in the electronic supplementary material.

A mathematical or computational model always represents a simplified reflection of the real complex system. Even if we build increasingly complex models, we will never completely approach reality. However, this poses no problem because we already have the real system. Restricting ourselves to capture only the essential processes in mathematical equations helps us to understand and predict the system's behaviour. Even if models gradually become quite complex, we can still track all interactions and how they synergize to yield the overall result. The easy accessibility of variables in computer models provides an advantage over the real system. Computational modelling is, therefore, a powerful tool to integrate all the knowledge of human scientists gathered in studies that, by themselves, consider only parts and details of the system. By doing this, we also produce powerful tools to predict the performance of the human body and learn how human body function may be improved in health and disease.

## 9. Conclusions

We conclude that simulation of athletic performance with a computational model of energy turnover and heat transport in the whole human body is feasible. The maximal oxygen uptake capacity of the athlete can be estimated based on performance in a race. The temperatures in various parts of the body such as brain and active muscle groups can be predicted. The changes in the phosphocreatine energy buffer status can be predicted, although glycolytic ATP production and pH regulation will have to be added. The model of carbon flux distribution in central metabolism allows us to investigate hypotheses on the apparent dysbalance of glucose and lactate uptake with cerebral metabolic needs.
